# Adventitial Niches, Complement and Inflammation in Pulmonary Vascular Disease: Current Status and Future Directions

**DOI:** 10.1002/cph4.70133

**Published:** 2026-03-26

**Authors:** Hui Zhang, Ram Raj Prasad, Sushil Kumar, Min Li, Dallas Jones, Cheng‐Jun Hu, Claudia Mickael, Yen‐Rei Yu, Rubin M. Tuder, Kurt R. Stenmark

**Affiliations:** ^1^ Department of Pediatrics and Medicine Cardiovascular and Pulmonary Research Laboratroy (CVP) University of Colorado Anschutz Medical Campus Aurora Colorado USA; ^2^ Department of Craniofacial Biology University of Colorado, Anschutz Medical Campus Aurora Colorado USA

## Abstract

There is strong evidence supporting inflammatory and autoimmune processes in the pathogenesis of pulmonary arterial hypertension (PAH), although the initiating and disease‐sustaining mechanisms remain unclear. Studies in arthritis, kidney disease, and cancer have demonstrated that dysregulation of the complement system can drive inflammation‐mediated tissue injury. We have shown that activation of the complement cascade, particularly the alternative pathway, within the pulmonary vasculature is a key driver of proinflammatory responses in pulmonary hypertension (PH). Single‐cell spatial transcriptomic analysis of PAH lungs further revealed complement‐rich adventitial fibroblasts, granzyme K (GZMK)^+^ CD8 T cells, and activated macrophages forming proinflammatory niches within the pulmonary artery adventitia in the different pulmonary vascular lesions in PAH. Our recent work highlights the role of both extracellular and intracellular complement activation in fibroblast‐mediated pulmonary vascular inflammation. Specifically, activation of the alternative pathway within fibroblasts, through complement factors D (CFD) and B (CFB), promotes production of anaphylatoxins (C3a and C5a), cytokines, and complement‐containing extracellular vesicles (EVs). These mediators drive macrophage and T‐cell recruitment and activation, contributing to disease progression. Additionally, a fourth pathway of complement activation mediated by CD8^+^ T cell–derived GZMK, which triggers extracellular complement cascades in fibroblasts and macrophages, has recently been identified. These findings support the hypotheses that local complement production by pulmonary artery adventitial fibroblasts, activated intracellularly by CFD and CFB and extracellularly by GZMK^+^, and its secretion in soluble form and within EVs promotes macrophage chemotaxis and activation. These complement‐driven proinflammatory niches in PAH pulmonary arteries represent promising therapeutic targets in PH.

## Graphical abstract

Local complement production by pulmonary artery adventitial fibroblasts, activated intracellularly by CFD and CFB and extracellularly by GZMK^+^ CD8 T cells, and its secretion in soluble form and within EVs promotes macrophage and T cell chemotaxis and activation. These complement‐driven proinflammatory niches in PAH pulmonary arteries represent promising therapeutic targets in PH.

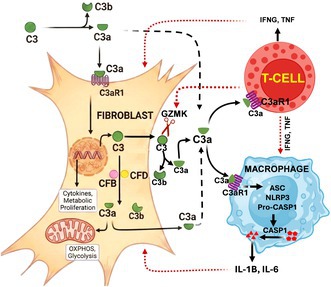

## 1 Introduction

Pulmonary vascular diseases (PVD) represent a devastating and heterogeneous spectrum of disorders unified by the presence of pulmonary hypertension (PH), a progressive condition marked by increased pulmonary vascular resistance, right ventricular failure, and premature death (Humbert et al. 2019; Pullamsetti et al. 2016; Guignabert et al. 2024). The epidemiological burden is substantial. PVD affects approximately 1% of the global population, and prevalence rises to nearly 10% in individuals older than 65 years (Hoeper et al. 2016; Collaborators GBDPAH 2025). Moreover, many more individuals harbor subclinical pulmonary vascular dysfunction, predisposing them to progression toward overt disease. The presence of PH, regardless of underlying etiology, confers increased morbidity and mortality across diverse patient groups. This broad impact highlights the need for a unified understanding of shared pathogenic mechanisms across etiologically distinct forms of PVD.

Despite decades of research and therapeutic progress, PH remains incurable for most patients. Currently approved drugs, primarily pulmonary vasodilators, offer symptomatic improvement but fail to reverse disease progression or modify the underlying pathobiology. The investigation into the central role of unbalanced TGFβ/bone morphogenetic protein (BMP)/Activin signaling in PH led to the development of the activin‐binding drug Sotatercept, which has provided the first glimmer of disease modification. Yet, its limitations, including complex dosing, adverse effects, and applicability to a narrow patient population, underscore the pressing need for the development of new therapies that target novel mechanisms involved in the pathobiology of disease.

PVD is characterized by extensive pulmonary vascular remodeling, involving all segments of the pulmonary circulation. Large elastic pulmonary arteries undergo stiffening with an increase in collagen in the vascular media, and, with prolonged elevated pulmonary artery pressure, may develop atherosclerosis in the subintima region (Tuder et al. 2013). The most marked structural alterations occur, however, in the muscular and precapillary pulmonary arteries. The intima and subintima region undergoes thickening, with increases of up to 3‐fold in Pulmonary Arterial Hypertension (PAH) compared with control lungs (Stacher et al. 2012). Localized intima thickening is largely due to the increase in myofibroblastic‐like cells and extracellular matrix, and can be found in normal lungs; more widespread intima thickening is found in all forms of PH. In the more severe group 1 PAH, total lumen obliteration occurs due to the presence of obliterative and plexiform lesions (Jonigk et al. 2011).

Medial hypertrophy (MH), defined by increased media thickness due to a higher number and increased size of pulmonary arterial smooth muscle cells, is observed across all forms of PH (Pietra et al. 2004). In normal pulmonary arteries, the medial layer accounts for approximately 4%–5% of the total vessel diameter, whereas in PH this proportion typically exceeds 10% of the diameter of hypertensive pulmonary arteries. Adventitial thickening in PAH was formerly reported to consist of an approximately 30% increase in adventitial fractional thickness compared with control vessels (Chazova et al. 1995).

While it is clear that PVD is a pan‐pulmonary artery disease that involves all three pulmonary vascular layers, recent digital spatial profiling (DSP) of up to approximately 19,000 transcripts in idiopathic PAH (IPAH) lesions, as compared to control pulmonary arteries, indicated that the IPAH adventitia and plexiform lesion harbored the most differentially expressed genes when compared with control pulmonary arteries and vs. the smooth muscle‐cell‐rich IPAH obliterative lesion and media hypertrophy (Tuder et al. 2024). These differentially expressed genes indicated an enrichment of several inflammatory pathways—including TNFα and interferon signaling—allied to pathways classically involved in PVD, such as TGFβ signaling and epithelial‐mesenchymal transformation (EMT), extracellular matrix remodeling, and hypoxia. These findings are consistent with the observation of inflammation in PVD, with the adventitia‐based inflammation correlating with pulmonary hemodynamic measurements in IPAH cohorts.

An extensive body of work has established that immune dysregulation, leading to chronic inflammation, is a central driver of pulmonary vascular remodeling and PH development. Immune cell accumulation, particularly macrophages and T cells, in the pulmonary vascular adventitia is observed across the spectrum of PVD, suggesting a key role for the adventitia in integrating immune signals in PVD (Stacher et al. 2012; Huertas et al. 2020; Mickael and Graham 2019; Nader et al. 2020; Sundd et al. 2019; Stenmark et al. 2013; Savai et al. 2012; Rabinovitch et al. 2014). Thus, the critical importance of the understanding of immune dysregulation in Pulmonary Hypertension (PH) lies on its role as a central driver of the vascular remodeling and disease progression, thus contributing to the devastating nature of the disease. Recently high‐throughput technologies, such as multiplexed ion beam imaging by time of flight (MIBI‐TOF), and integration of multiplexed fluorescent in situ hybridization (FISH) with high‐throughput imaging of single‐cell spatial profling at a microscopic level have opened new frontiers in this pursuit by enabling the simultaneous study of multiple immune cells, their activation states, and spatial arrangements within lung tissue (Zhao et al. 2025; Ferrian et al. 2024). Using MIBI‐TOF (Ferrian et al.) imaged a comprehensive panel of 35 proteins—spanning immune cell differentiation and function alongside tissue‐specific markers to generate an architectural map of PAH lung tissue, directly linking immune cell subsets to pulmonary arterial (PA) lesions. This unprecedented mapping underscores offered an unprecedent insight into the complexity and immune architecture in the PAH pathology. Interestingly these approaches allow comparisons of cell types and activation states in different forms of PH. For instance Hereditary PAH (HPAH) reveals a striking pattern of heightened perivascular inflammation, compared to IPAH, supporting the importance of investigating immune disorders specific different subtypes of PH.

**FIGURE 1:**
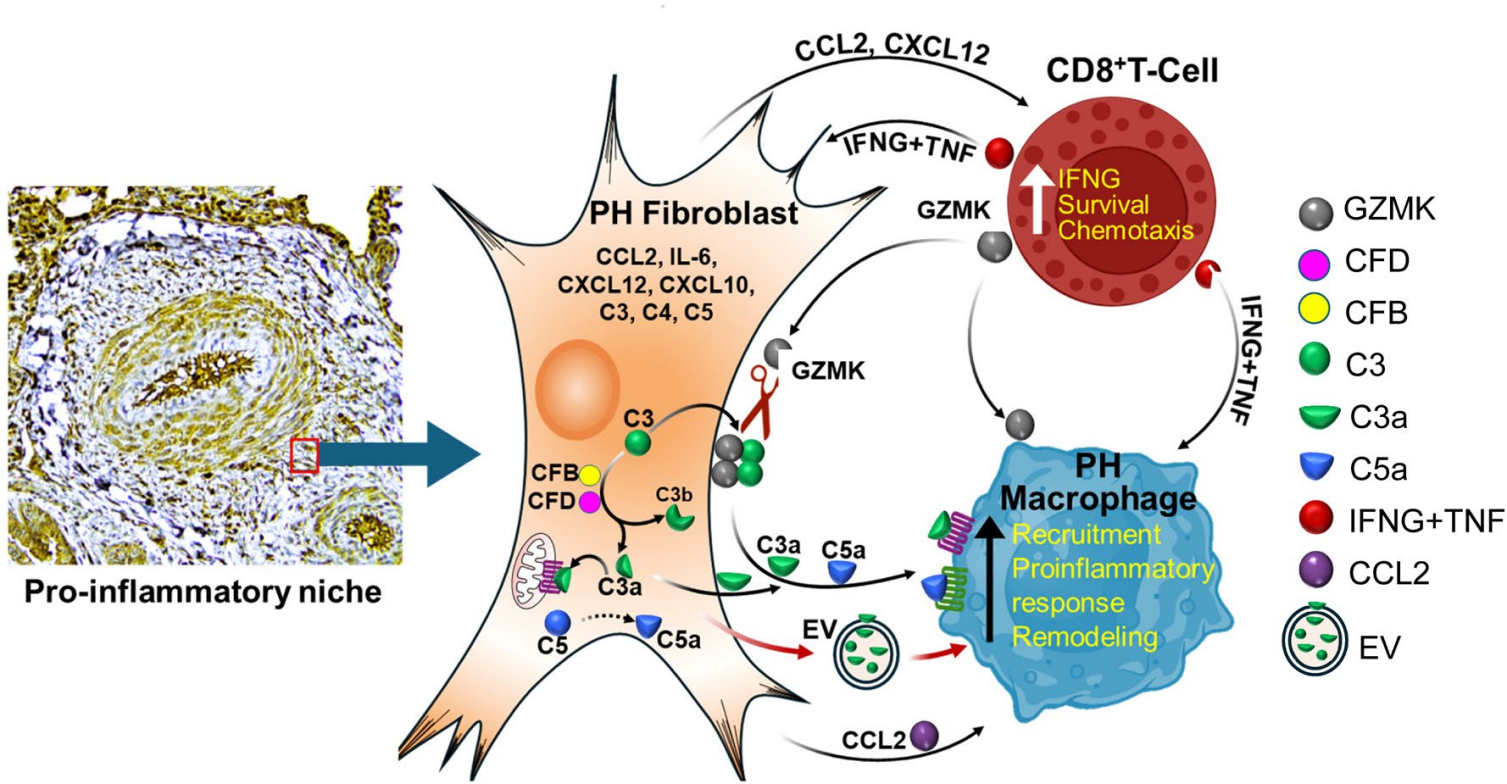
Hypothesis: Complement–cytokine crosstalk and immune amplification within the pulmonary artery adventitial proinflammatory niche.

Research over the past decade has increasingly supported the concept that the vascular adventitia acts as a biological processing center for the retrieval, integration, storage, and release of key regulators of vessel wall function (Zou et al. 2025). In addition to fibroblast cells, the pulmonary vascular adventitia comprises a variety of cell types, including resident progenitor cells, vasa vasorum endothelial cells, adrenergic nerves, and a variety of immune cells, including macrophages, T‐cells, B‐cells, dendritic cells, and NK‐cells (Huertas et al. 2020; Stenmark et al. 2013; Duffield et al. 2013; Majesky et al. 2011; Tuder 2017). Leveraging published insights into the cellular and molecular proinflammatory and pro‐remodeling hubs centered within and around the PH adventitia cell populations in PVD, we propose a hypothesis that these PAH adventitia cell populations are largely coordinated by the adventitial fibroblasts, leading to the organization of these cellular and molecular actors in proinflammatory niches. These adventitial niches involve cross interaction with macrophages, T‐, and B‐ cells, which promote molecular signaling leading to persistent inflammation and PVD (Figure 1). We suggest that epigenetically imprinted pathogenic fibroblast cell states drive persistence of inflammation through complement activation. We support this concept with published and pilot data indicating that complement activation, triggered by adventitial fibroblasts is central in the formation and molecular characterization of these inflammatory niches. We discuss therapeutic strategies that could be explored to specifically treat pathogenic subsets of fibroblasts and their interactions with inflammatory cells in PH.

## 2 Vascular Adventitial Proinflammatory Niches Drive Inflammation in PVD

**FIGURE 2:**
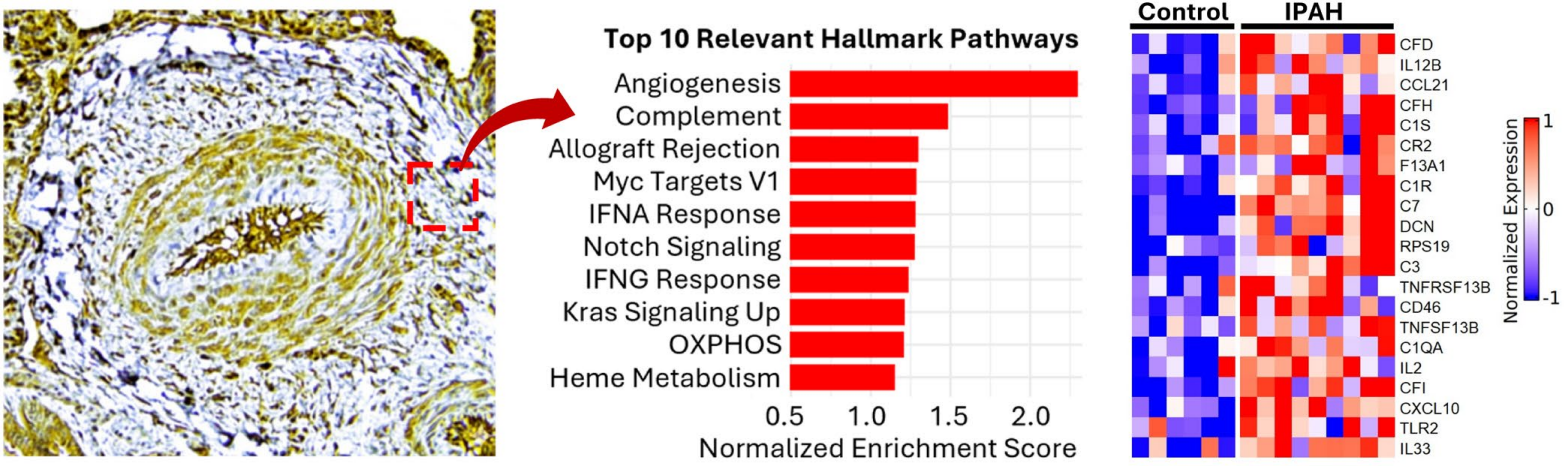
Digital spatial profiling of IPAH pulmonary artery adventitia. Top 10 hallmark pathways enriched in IPAH adventitia when compared to control. Heatmap depicting complement genes in control and IPAH pulmonary artery adventitia. (Adapted from Ref (Tuder et al. 2024)).

The role of pulmonary vascular adventitia in promoting inflammation and vascular remodeling is supported by modern molecular spatial technologies at single‐cell and subcellular resolution, which have revolutionized our ability to interrogate cells and processes at the microscopic level, within histological samples (Zhao et al. 2025; Ferrian et al. 2024; Cober et al. 2025; Tsukui et al. 2024; Melms et al. 2021). Using these technologies, it is now recognized that significant cellular and molecular heterogeneity exhists in normal versus pulmonary vascular diseased tissues (Tuder et al. 2024; Ferrian et al. 2024; Sikkema et al. 2023). Spatial technologies have further allowed interrogation of various vascular niches that can exist in the setting of PH. A recent study using DSP of IPAH lesions significantly strengthens this concept (Tuder et al. 2024). In this study, plexiform lesions, concentric neointimal lesions, and adventitial regions were profiled using approximately 19,000 genes per microscopic lesion, defined as regions of interest (ROI). Adventitial ROIs uniquely exhibited strong enrichment for inflammatory response signatures, dramatic upregulation of the complement system (C3, CFB and CFD), and increased antigen presentation and lymphocyte‐activating modules, all within a fibroblast‐dominant stromal architecture (Figure 2). These findings established the adventitia as perhaps the most immunological and complement‐rich region in IPAH and support the hypothesis that local complement production, activation and secretion, are involved in the formulation and sustenance of inflammatory niches (Tuder et al. 2024).

**FIGURE 3:**
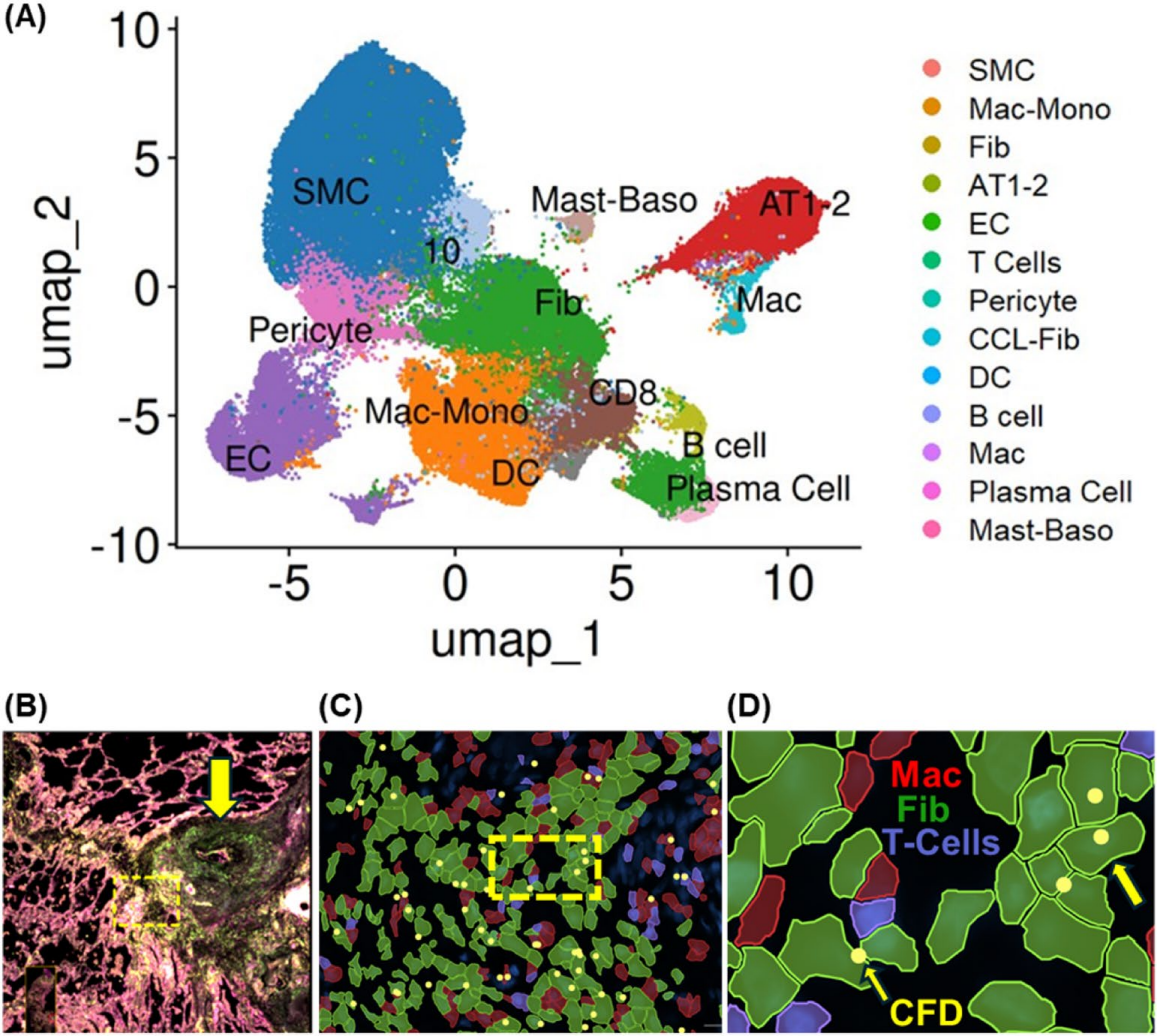
Dominant CFD expression in IPAH fibroblasts. (A) Cell clusters identified in the 10 pulmonary arteries of an IPAH lung. Cell identifiers within the pulmonary arteries (defined as regions of interest (ROI)) were used to subset the approximately 10^6 cells scanned by the 10× Xenium platform, resulting in approximately 300,000 cells in these ROIs. Cell identities were attributed based on the expression of characteristic transcripts, as based on literature review, including the lung cell atlas. (B) Low magnification of IPAH pulmonary artery with a plexiform lesion (arrow). (C) Plexiform lesion adventitia (dashed box in C) with adventitia fibroblasts (Fib, green), macrophages (Mac, red) and CD8 T cells (purple). (D) CFD (yellow dots, highlighted with yellow arrows) found mainly in fibroblasts in IPAH adventitia from B (dashed box).

DSP, while providing groundbreaking data on the transcriptomic architecture of microscopic structures, is limited by its inability to define transcript expression at a single‐cell level. In pilot experiments using single‐cell spatial transcriptomic profiling, we examined an IPAH lung sample with 10 pulmonary arteries with characteristic IPAH lesions—plexiform, obliterative, and intima‐media hypertrophy—surrounded by distinct adventitial regions from a single representative IPAH lung and then compared with control pulmonary arteries in a single normal lung analyzed. This consisted of approximately 90,000 cells from the IPAH lung and 11,500 cells from the control. This pilot study demonstrated the enrichment and aggregation of fibroblasts, macrophages, T cells, and complement (as exemplified by complement factor D, CFD) within the adventitial space (Figure 3). Using analytical bioinformatic pipelies shared with standard single‐cell transcriptomic analyses for dissociated cells (such as the R package Seurat), we found an extensive heterogeneity in immune and fibroblast populations, revealing multiple previously unrecognized fibroblast, endothelial, and macrophage cellular subsets (data not shown). These observations are consistent with those of Zhao et al. (2025) and Ferrian et al. (2024).

While disease triggers differ across the spectrum of PVD, ranging from idiopathic pulmonary vascular disease to cardiopulmonary and other associated diseases, immune activation with the accumulation of macrophage and T cells in the fibroblast‐rich adventitial microenvironment has been largely well‐documented in PAH (Guignabert et al. 2024; Stacher et al. 2012; Savai et al. 2012; Rabinovitch et al. 2014). However, PH in the setting of chronic obstructive pulmonary disease (COPD), systemic sclerosis (SSc) and pulmonary fibrosis occurs within a rich inflammatory mileu, likely also involving the pulmonary vascular adventitia. These findings are consistent with the concept that the adventitia acts as a command center and the adventitial fibroblast as a key cellular/molecular relay in regulating vascular homeostasis and, when dysregulated, orchestrating pathological remodeling (Huertas et al. 2020; Stenmark et al. 2013; Zou et al. 2025; Tuder 2017; Zhang et al. 2024). However, despite ample data supporting the central role of the pulmonary vascular adventitia in PVD pathogenesis, the cellular composition and molecular processes within the adventitial space remain poorly understood. Based on experimental data, we hypothesize that in PAH, there is a convergence of distinct adventitial fibroblast populations that sustain pro‐inflammatory and pro‐remodeling adventitial niches, cross‐regulating macrophage and T cell activation, and propagating inflammation (Figure 1—shown above) (Tuder et al. 2024). However, distinct adventitial fibroblasts and immune cell populations likely exist in different forms of PVD, induced by distinct inciting conditions but converging in pro‐remodeling processes that promote PH.

## 3 Fibroblasts as Central Orchestrators of the Pro‐Inflammatory and Pro‐Remodeling Adventitial Niche

Fibroblasts, once viewed simply as structural cells primarily involved in tissue integrity and anatomical compartmentalization via their actions on the extracellular matrix, are now recognized as highly dynamic regulators of not only tissue homeostasis but also immune function (Zou et al. 2025; Tsukui et al. 2024; Cowan et al. 2025; Li, Wu, et al. 2021; Zhang, Jonsson, et al. 2023). In health, fibroblasts shape tissue architecture by synthesizing and remodeling extracellular matrix (ECM), organizing cellular microenvironments (or niches), and directing immune cell positioning, activation, and survival. Specialized fibroblast populations, such as fibroblastic reticular cells (FRCs) in lymphoid tissues, actively orchestrate adaptive immune responses by guiding lymphocyte trafficking, antigen presentation, and the balance between immune activation and resolution (Li, Wu, et al. 2021). Through spatially restricted secretion of chemokines, cytokines, and morphogens, fibroblasts define the functional organization of tissues across diverse organs, including lymph nodes, synovium, gut, skin, and lung.

In diseases such as rheumatoid arthritis (RA) and pulmonary fibrosis, fibroblasts undergo profound functional reprogramming (Tsukui et al. 2024; Zhang, Jonsson, et al. 2023; Zhang et al. 2019). Rather than resolving inflammation or restoring tissue integrity, they can adopt inflammatory, fibrogenic, tissue‐degradative, or proliferative phenotypes that actively drive disease development. In chronic inflammatory diseases such as RA, fibroblasts become dominant producers of pro‐inflammatory cytokines and chemokines, recruit and retain leukocytes, and form tertiary lymphoid structures that sustain autoreactive immune responses (Zhang et al. 2019). In parallel, distinct fibroblast subsets acquire invasive and matrix‐degradative programs, directly enacting cartilage and bone destruction. In fibrotic diseases such as pulmonary fibrosis, persistent myofibroblasts deposit excessive and disorganized ECM, whereas in cancer, specialized cancer‐associated fibroblasts promote immune evasion, tumor growth, and therapeutic resistance. Across these contexts, fibroblast expansion and persistence are closely linked to poor prognosis and resistance to current immunomodulatory therapies (Zou et al. 2025; Davidson et al. 2021).

Single‐cell studies of enriched fibroblast organ populations and the use of microscopic tissue transcriptomic and protein spatial profiling have fundamentally transformed the understanding of fibroblast biology by revealing extensive transcriptional and functional heterogeneity within and across tissues (Vannan et al. 2025; Tian et al. 2023). Diseased tissues contain multiple fibroblast subpopulations with distinct functions, including inflammatory and immune‐interacting, matrix‐remodeling, migratory or tissue invasive, vascular‐interacting, and progenitor‐like states (Zou et al. 2025; Vannan et al. 2025). Some fibroblast programs are tissue‐specific, reflecting positional identity and local niche signals, whereas others are conserved across organs and diseases, indicating shared pathogenic pathways. In RA and other chronic inflammatory diseases, inflammatory and vascular‐interacting fibroblasts dominate leukocyte‐rich regions, while matrix‐remodeling or invasive fibroblasts localize to zones of tissue destruction. Importantly, fibroblast‐rich tissue pathotypes are strongly associated with failure to respond to biologic and targeted therapies, underscoring their clinical relevance (Zou et al. 2025; Davidson et al. 2021).

The differentiation and activation of pathogenic fibroblast states are driven by an interplay of cell‐extrinsic and intrinsic mechanisms. Spatially organized morphogen gradients, such as Notch, Wnt, BMP, and Hedgehog signaling, establish positional identity and zonated fibroblast phenotypes within tissues (Zhang, Jonsson, et al. 2023). Pro‐inflammatory cytokines—including TNF, IL‐1β, IL‐17, interferons, and oncostatin M—act synergistically to induce inflammatory programs, which are then sustained by autocrine amplification loops involving IL‐6, LIF, and related pathways (Smith et al. 2023; West et al. 2017). Adhesion molecules and mechanotransduction pathways enable fibroblasts to sense and respond to ECM stiffness and tissue injury, shaping inflammatory, invasive, or fibrogenic behaviors. These signals are further stabilized by epigenetic reprogramming, including DNA hypomethylation and activating histone modifications at cytokine, chemokine, and matrix‐remodeling gene loci, creating an “inflammatory memory” that persists even outside the diseased tissue environment.

**FIGURE 4:**
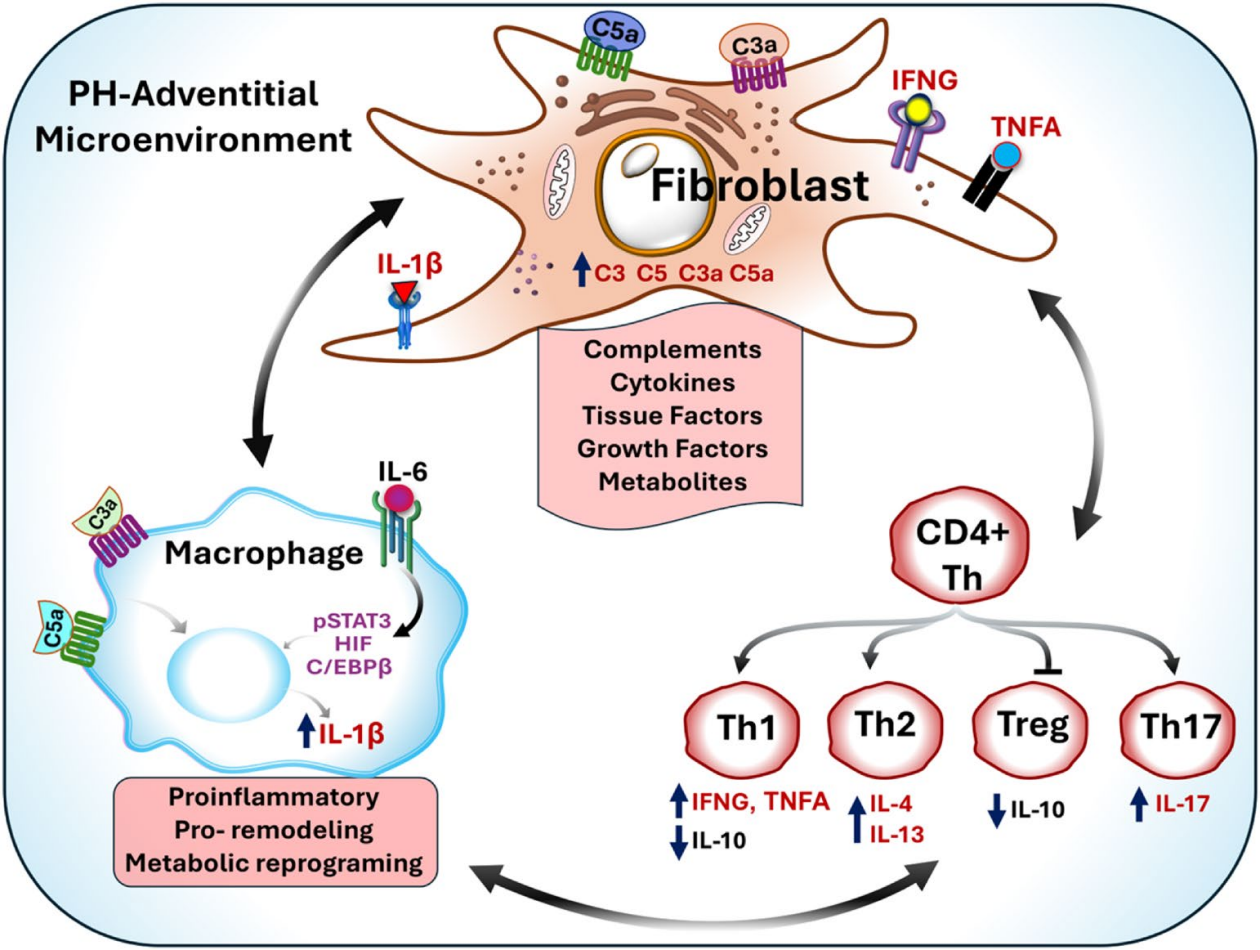
Crosstalk among fibroblast, myeloid, and lymphatic in PH‐adventitial microenvironment.

**FIGURE 5:**
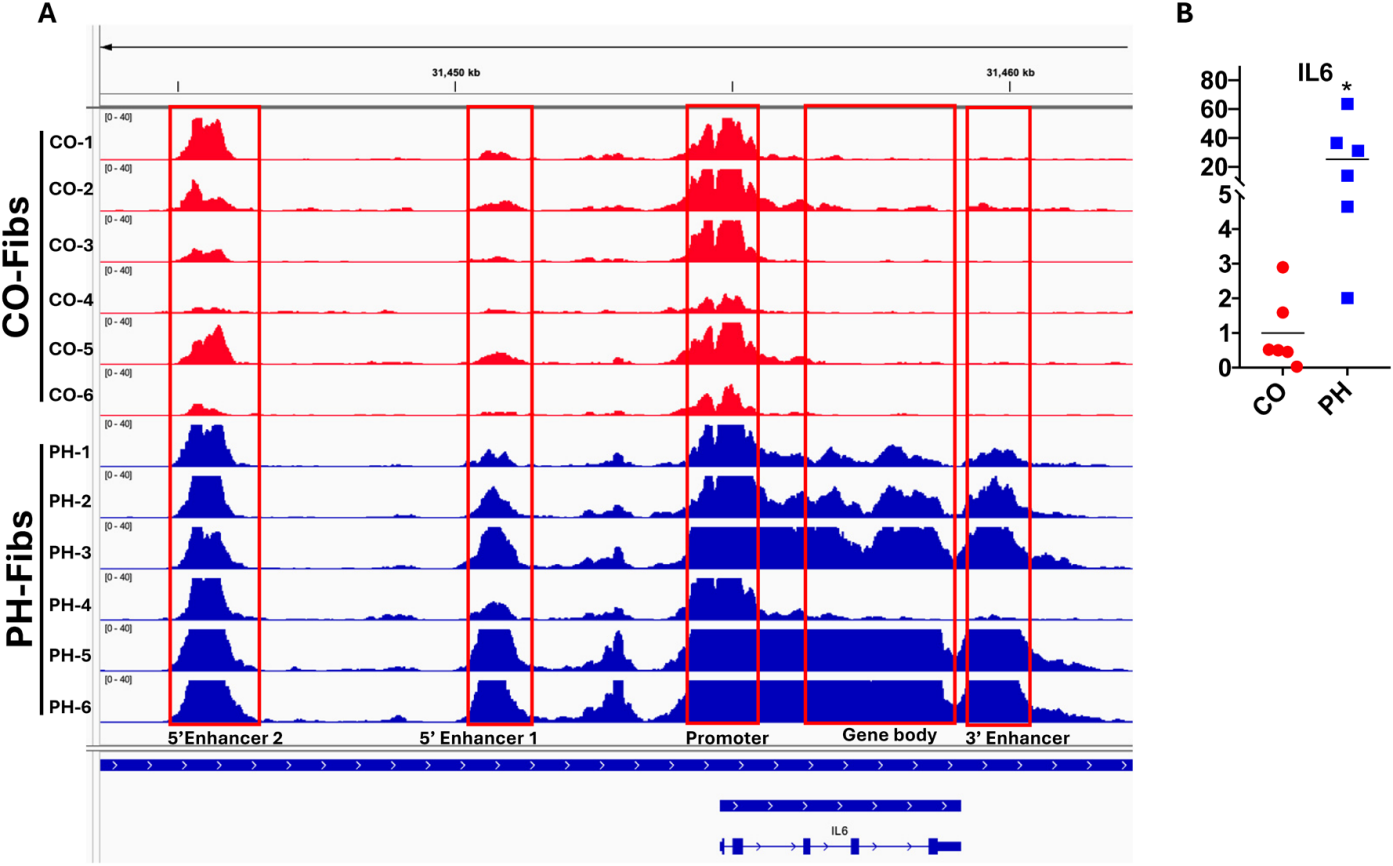
Increased inflammatory gene expression is correlated with activated (open) chromatin in pulmonary artery adventitia fibroblasts, indicated by integrated ATAC‐seq and RNA‐seq. Profile of DNA accessibility and gene expression for IL6 in CO‐Fibs (red) and PH‐Fibs (blue) (*n* = 6 each). A, ATAC‐seq data indicate the chromatin structure of IL6 genes is more open in PH‐Fibs compared to CO‐Fibs (in enhancers, promoters and/or gene body region). The “openness” of IL6 genes, are paralleled with the increased gene expression in PH‐Fibs (B, RNA‐seq).

Consistent with observations regarding fibroblasts in other chronic inflammatory diseases, pulmonary arterial adventitial fibroblasts have been identified as exhibiting the earliest, most dramatic, and most sustained proliferative, apoptosis‐resistant, fibrotic, and inflammatory responses to vascular stress, thereby playing a pivotal role in the progression of PH (Stenmark et al. 2013; Duffield et al. 2013; Majesky et al. 2011; Price et al. 2012; Belknap et al. 1997; Meyrick and Reid 1980; Stenmark et al. 2006; Alexanian et al. 2023; Gangishetti et al. 2020; Krosel et al. 2023; Aguado‐Alvaro et al. 2024). Under homeostatic conditions, four main types of lung fibroblasts have been identified based on their structural location: alveolar, peribronchial, subpleural, and adventitial fibroblasts (Sikkema et al. 2023). While all these populations exhibit shared transcripts such as COL1A2, DCN, and MFAP4, adventitial fibroblasts can be identified by enhanced expression of MFAP5, SCARA5, PI16, and CCL11 and represent a unique fibroblastic cell type. PH fibroblasts exhibit excessive proliferation, resistance to apoptosis, metabolic reprogramming, and altered secretory activity, underscoring their role in dramatically activating inflammatory signaling in macrophages and lymphocytes (Stenmark et al. 2013; Zhang et al. 2024; Kumar et al. 2021; Plecita‐Hlavata et al. 2023; Buckley et al. 2015; Goldenberg et al. 2019; Li et al. 2016; Tieu et al. 2009) (Figure 4). The persistently activated phenotypic changes in adventitial fibroblasts are sustained over multiple passages in culture ex vivo and in the absence of an in vivo environment (Li et al. 2011). It should be noted that it appears that fibroblasts involved undergo marked epigenetic changes as shown with other inflammatory diseases. By ATACseq and RNAseq, we observed changes consistent with an open chromatin structure in many inflammatory gene transcription control sites, which would enable feedforward inflammatory signaling (Figure 5).

The inability of activated fibroblasts to revert to a quiescent state, even in vitro, suggests a cell‐autologous role of PH fibroblasts in driving chronic inflammation and pulmonary vascular remodeling in PH (Duffield et al. 2013; Buckley et al. 2001). Consistent with their role in driving the pulmonary vascular inflammation and remodeling in PH, in both IPAH and animal models of PH, the PH fibroblast phenotype is characterized by heightened expression of proinflammatory cytokines/chemokines (GM‐CSF, CCL2, CX3CL1, CXCL12, IL6) and prominent expression of complement components (e.g., C3, CFB, and CFD), which can in turn induce activation and pro‐inflammatory programming in macrophages and T cells.

### 3.1 Complement Pathways Shape the Pro‐Inflammatory Adventitial Microenvironment in PH

**FIGURE 6:**
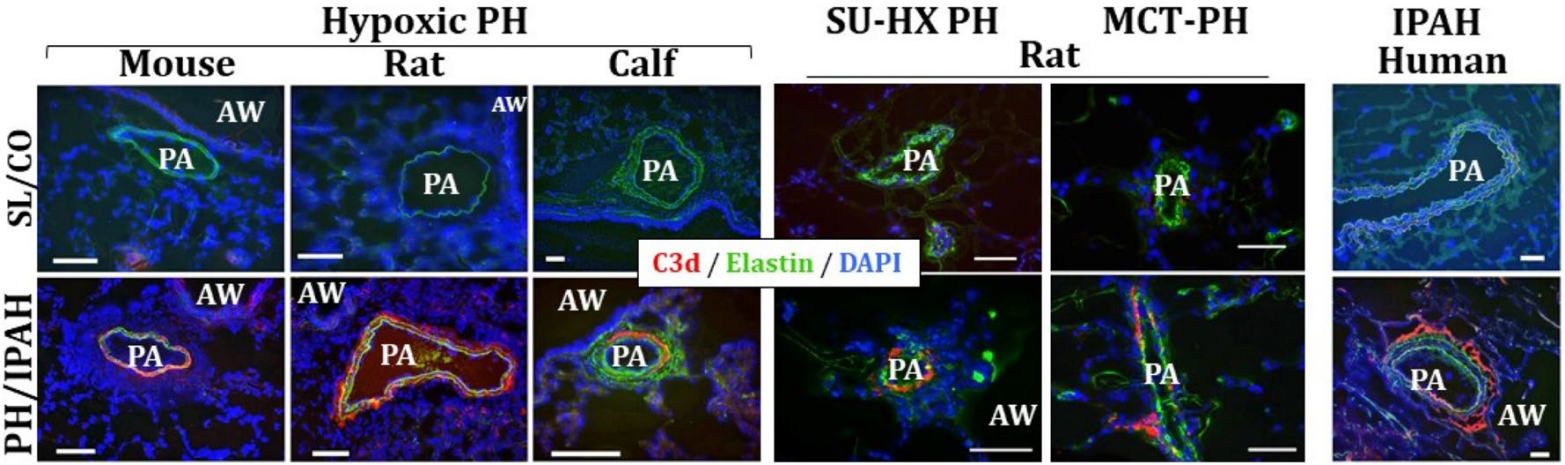
Activation of the complement cascade, as defined by deposition of C3d (terminal degradation fragment of C3 activation) is observed in a perivascular‐specific pattern in the lungs of experimental animal models of PH (3‐week hypoxic mice and rats; 2‐week hypoxic calves; and rats with sugen‐hypoxia (SU‐HX)‐ or monocrotaline (MCT)‐induced PH) and in humanswith idiopathic PAH (IPAH). No deposition of C3d is observed in normal control lungs. Cryosectiones were immunolabeled with C3d‐specific monoclonal antibody mAbs‐C3d29 (red fluorescence), which distinguishes tissue‐bound C3d from C3 or C3b. Pulmonary arteries (PAs) are visualized by autofluorescence of elastic lamellae (green). AW, airways. Cell nuclei are labeled with DAPI (blue fluorescence). CO, control; SL, sea level. Scale: 100 mm. (Adapted from ref. (Frid, Thurman, et al. 2020)).

The complement system has a significant role in regulating the development and progression of PH by promoting inflammation and vascular remodeling (Tuder et al. 2024; Frid, McKeon, et al. 2020; Frid, Thurman, et al. 2020). Our recent work confirmed increased accumulation of complement proteins in both large and small pulmonary arteries, further implicating complement as a participant in PH pathobiology (Williams et al. 2024). Collectively, sub‐cellular resolution spatial transcriptomics and immunohistochemical analyses showed that complement signaling is prominently observed in the adventitial space of IPAH tissues, supporting a significant role for adventitia in regulating a pro‐inflammatory and pro‐remodeling microenvironment via complement activation (Tuder et al. 2024; Frid, McKeon, et al. 2020; Frid, Thurman, et al. 2020) (Figure 6).

**FIGURE 7:**
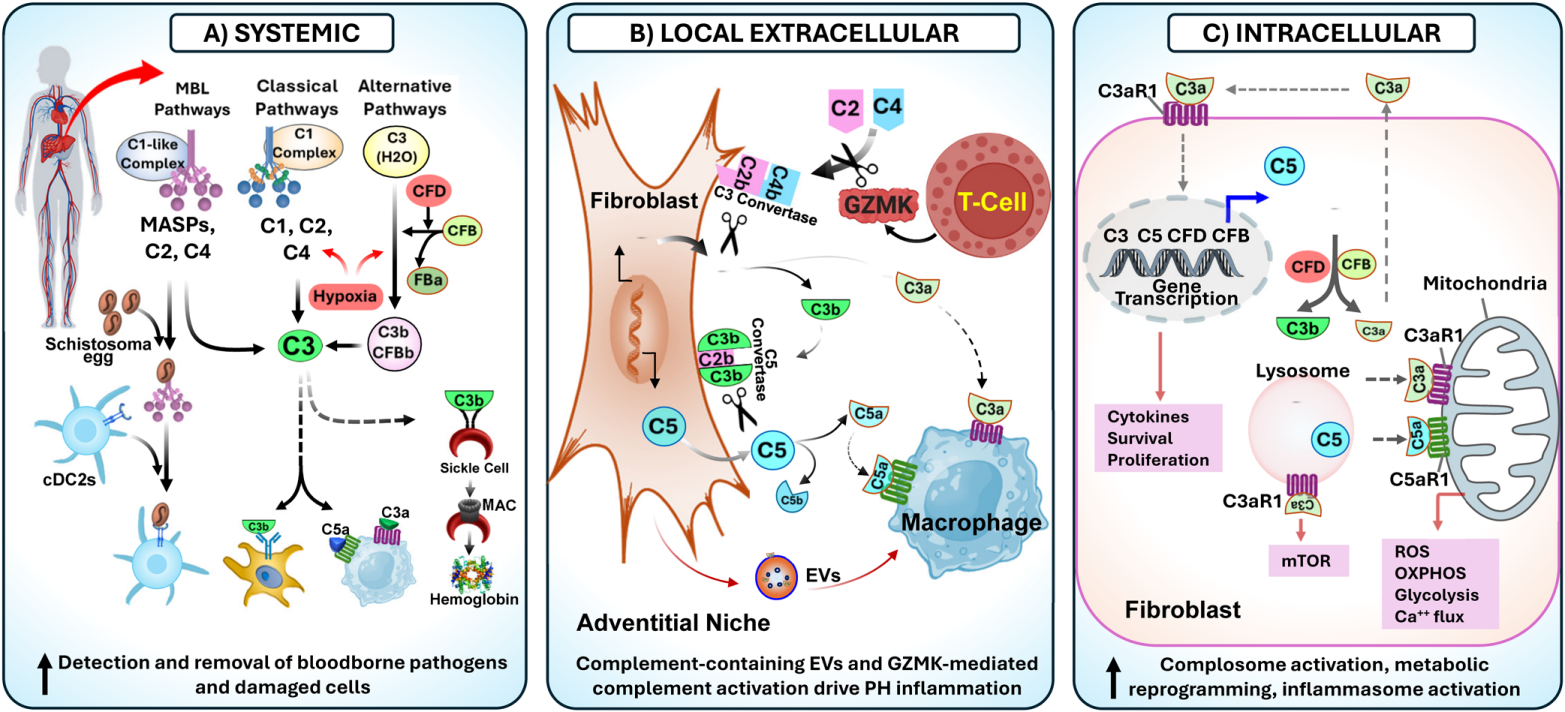
Overview of systemic (A), local extracellular (B), and intracellular (C) complement production and activation by the liver, pulmonary artery, and fibroblasts, emphasizing their roles in clearing pathogens and damaged cells, driving inflammation, and regulating metabolism.

Complement is a conserved component of the innate immune system. Complement activation involves a multi‐step process with sequential proteolytic steps through three convergent pathways (classical, lectin, and alternative pathways), ultimately leading to active membrane attack complex C5b‐9 (Kemper et al. 2023; Pouw and Ricklin 2021; Ricklin et al. 2010; Mastellos et al. 2024). The classical and lectin pathways involve a triphasic sequence of recognition, initiation, and execution. Soluble pattern recognition receptors, such as C1q (in the classical pathway of complement activation) and mannose‐binding lectin (MBL, in the lectin pathway), spearhead this process by detecting immunoglobulin and danger signals on target surfaces, respectively. This recognition triggers activation of initiator proteases (C1s and MASP1, respectively), which cleave C4 and C2, leading to the assembly of C3 and C5 convertases (Figure 7A). The alternative pathway requires complement factor B (CFB) and CFD to generate the C3 convertase, which can, in turn, generate the C5 convertase. These convertases drive the execution phase, cleaving C3 and C5 to create the effector molecules of the complement cascade (anaphylatoxins and convertases). In the context of PVD, we showed that complement activation, involving C3 and C4, participates in the pro‐inflammatory and pro‐remodeling processes in PAH (Frid, McKeon, et al. 2020; Frid, Thurman, et al. 2020). We have also found that MBL activation of lectin pathways on CD4 T cells regulates the development of Schistosomasis associated PAH (Kumar et al. 2024). Additionally, activation of the alternative pathway induces hemolysis and tissue damage in sickle cell disease, contributing to the accumulation of heme and iron overload in macrophages, leading to the development of sickle cell disease associated PH (Redinus et al. 2019). These observations support a central role for three complement pathways in PVD/PH pathogenesis.

Complement signaling pathways have long been considered a liver‐derived, plasma‐based, immune surveillance system. Newer data, however, support a division of labor within the complement system, with circulating complement guarding the vascular space through its canonical antimicrobial functions. In contrast, locally cell‐derived complement regulates the phenotype of cells, including fibroblasts and immune cells in the microenvironment (Figure 7B) (Liszewski et al. 2013; West and Kemper 2023; Prasad et al. 2025; Maffia et al. 2024). Studies have shown that the compartmentalization of complement function extends to a third location—the interior of cells, which is known as the “complosome” (Liszewski et al. 2013; West and Kemper 2023; Prasad et al. 2025). The core components of the complosome, C3 and C5, along with their activation fragments (C3a, C5a) and receptors, as well as some complement regulators, have been detected in various intracellular locations, including the cytoplasm, lysosome, endoplasmic reticulum, mitochondria, and nucleus (Figure 7C). We and others have documented the presence of intracellular C3, CFB, and CFD in fibroblasts of PH vessels using scRNA‐seq and DSP in both small elastic vessels (1–3 mm) and distal small vessels (≤ 300 μm) in diameter (Tuder et al. 2024; Prasad et al. 2025; Chelladurai et al. 2022; Crnkovic et al. 2022). We have further shown that activation of the complosome in PH fibroblasts regulates metabolism and the production of complement and cytokines (Prasad et al. 2025).

**FIGURE 8:**
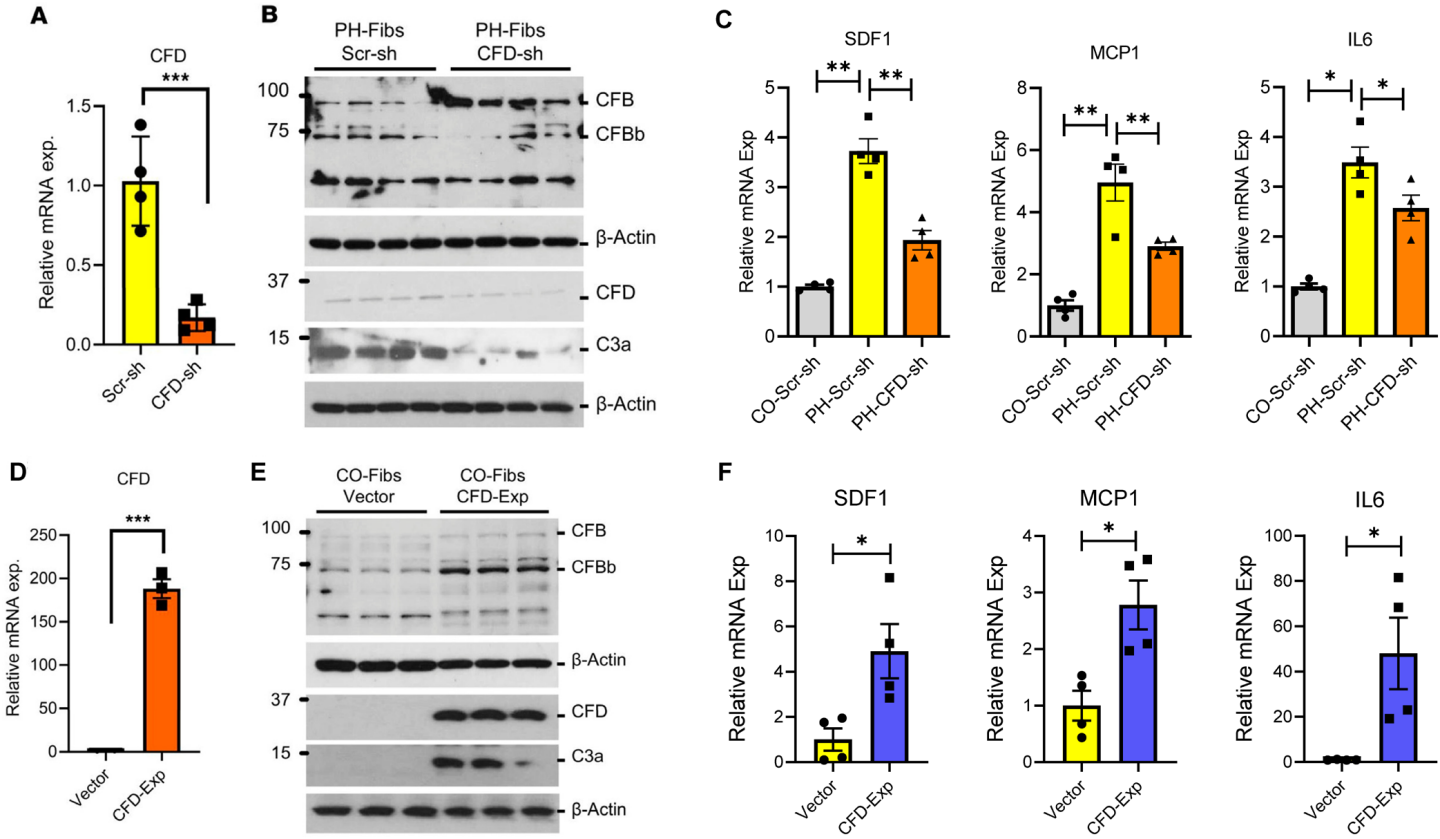
CFD regulates the activation of C3 in pulmonary artery adventitial fibroblasts and promotes the expression of inflammatory genes. (A, B) CFD knockdown results in decreased levels of CFB and C3 activation (C3a). (C) CFD knockdown in PH‐Fibs reduces levels of proinflammatory genes, including SDF1, MCP1, and IL‐6. (D, E) CFD overexpression in CO‐Fibs promotes CFB and C3 activation (C3a). (F) CFD overexpression increases the expression of SDF1, MCP1, and IL6 in CO‐Fibs. (Adapted from ref. (Prasad et al. 2025)).

Recent findings highlight a critical and previously underexplored role for intracellular complement proteins—the complosome—particularly C3, CFD, and CFB, in driving the metabolic and pro‐inflammatory reprogramming of hypertensive pulmonary artery fibroblasts (Prasad et al. 2025). Using single‐cell RNA sequencing of distal pulmonary arteries from humans and young bovine calves with severe PH, we identified significantly increased expression of C3, CFD, and CFB in fibroblasts isolated from PH subjects. Immunohistochemical analyses corroborated these findings at the protein level, demonstrating elevated CFD expression within the adventitial compartment of PAs from patients with IPAH (Tuder et al. 2024). Consistent with these in situ observations, fibroblasts cultured from PH vessels exhibited persistently elevated C3, CFD, and CFB mRNA expression, accompanied by a more accessible chromatin configuration at these loci compared with control fibroblasts. Importantly, we observed enhanced intracellular activation of complement components in PH fibroblasts, as evidenced by increased levels of the activation products C3a and CFBb relative to control cells. We further demonstrated expression of the C3a receptor, C3aR1, in adventitial fibroblasts, with significantly higher levels detected in IPAH‐derived cells. Subcellular fractionation studies revealed that C3aR1 is localized to both the plasma membrane and mitochondria, while complementary analyses identified mitochondrial accumulation of C3a itself (Prasad et al. 2025). In addition, C3a was localized to mitochondria and released into the extracellular space by fibroblasts (Prasad et al. 2025). Remarkably, CFD knockdown reverted the metabolic changes seen in PH‐Fibs, particularly normalizing the TCA cycle and fatty acid metabolism (Prasad et al. 2025). Furthermore, CFD‐mediated complement activation (C3a) was significantly positively correlated with the expression of inflammatory genes such as MCP1, SDF1, and IL‐6 (Figure 8) (Prasad et al. 2025).

Collectively, these findings establish the presence of a functionally active complosome within PH adventitial fibroblasts and support its role as a previously unrecognized mechanism contributing to pulmonary vascular inflammation and remodeling in PH. The specific contribution of local complement signaling to the PVD disease spectrum needs to be examined.

**FIGURE 9:**
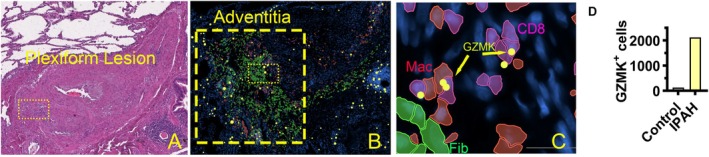
Spatial transcriptomics defines fibroblast‐ GZMK^+^ CD8 T cell‐ macrophage crosstalk in IPAH. Plexiform lesion shown in (A), with the adventitia area highlighted in the dashed box in (A) (hematoxylin and eosin) and the smaller box in (B) (Xenium DAPI and cell identity overlay) shown in higher magnification (C). The larger box in (B) highlights the overall adventitia in the plexiform lesion. (D) Quantification of GZMK^+^ CD8 T cells in adventitia between control and IPAH ROIs of similar size.

Additionally, the molecular signals leading to complement activation have recently been expanded by the discovery that granzyme K (GZMK) can directly promote cleavage and activation of C3 (Donado et al. 2025; Jonsson et al. 2022). The serine protease GZMK, primarily secreted by CD8 T cells and, to a lesser extent, by subsets of CD4 T cells and NK cells, bypasses classical pattern recognition to cleave complement substrates directly. In elegant studies of RA synovium, Johnson et al. demonstrated that GZMK binds heparan sulfate glycosaminoglycans on cell surfaces, enabling cleavage of C4 and C2 to form a C3 convertase independent of canonical initiators (Donado et al. 2025). Importantly, fibroblasts in RA lesions were the main producers of complement proteins serving as substrates for this pathway, positioning stromal–immune interactions at the center of this noncanonical cascade. These insights translate compellingly to PH. Spatial transcriptomic analyses of IPAH lungs revealed enrichment of GZMK within the adventitial region, precisely where fibroblasts, macrophages, and T cells aggregate (Figure 9). GZMK was co‐expressed with inflammatory transcripts, suggesting that CD8 T cells may act as rapid‐response effectors of local complement activation. This represents a paradigm shift: complement in PH is not solely the product of plasma proteins or fibroblast secretion but can be directly mobilized by cytotoxic lymphocytes at the vascular interface. Such a mechanism explains the persistence of inflammation in PH, as GZMK+ CD8 T cells are abundant in IPAH lungs (Ferrian et al. 2024; Li et al. 2024), and unlike other granzymes, GZMK is constitutively secreted without requiring T cell receptor engagement (Donado et al. 2025).

### 3.2 Propagation of Complement Mediated Inflammation via Extracellular Vesicles

A hallmark of vascular inflammation is dynamic intercellular communication through direct cell–cell contact or soluble paracrine mediators. However, there is increasing attention on the local effects of secreted extracellular vesicles (EVs) on target cells. EVs, which contain proteins and microRNAs, are now recognized as critical regulators of intercellular communication, tissue homeostasis, and pathological processes, as supported by numerous studies across multiple tissues, biological fluids, and cell types (Chan et al. 2019; Liu et al. 2021; Khandagale et al. 2020; Karasu et al. 2018; Barrachina et al. 2019; Mallia et al. 2020). However, whether EVs carrying complement components play a specific role in PH had remained unknown.

**FIGURE 10:**
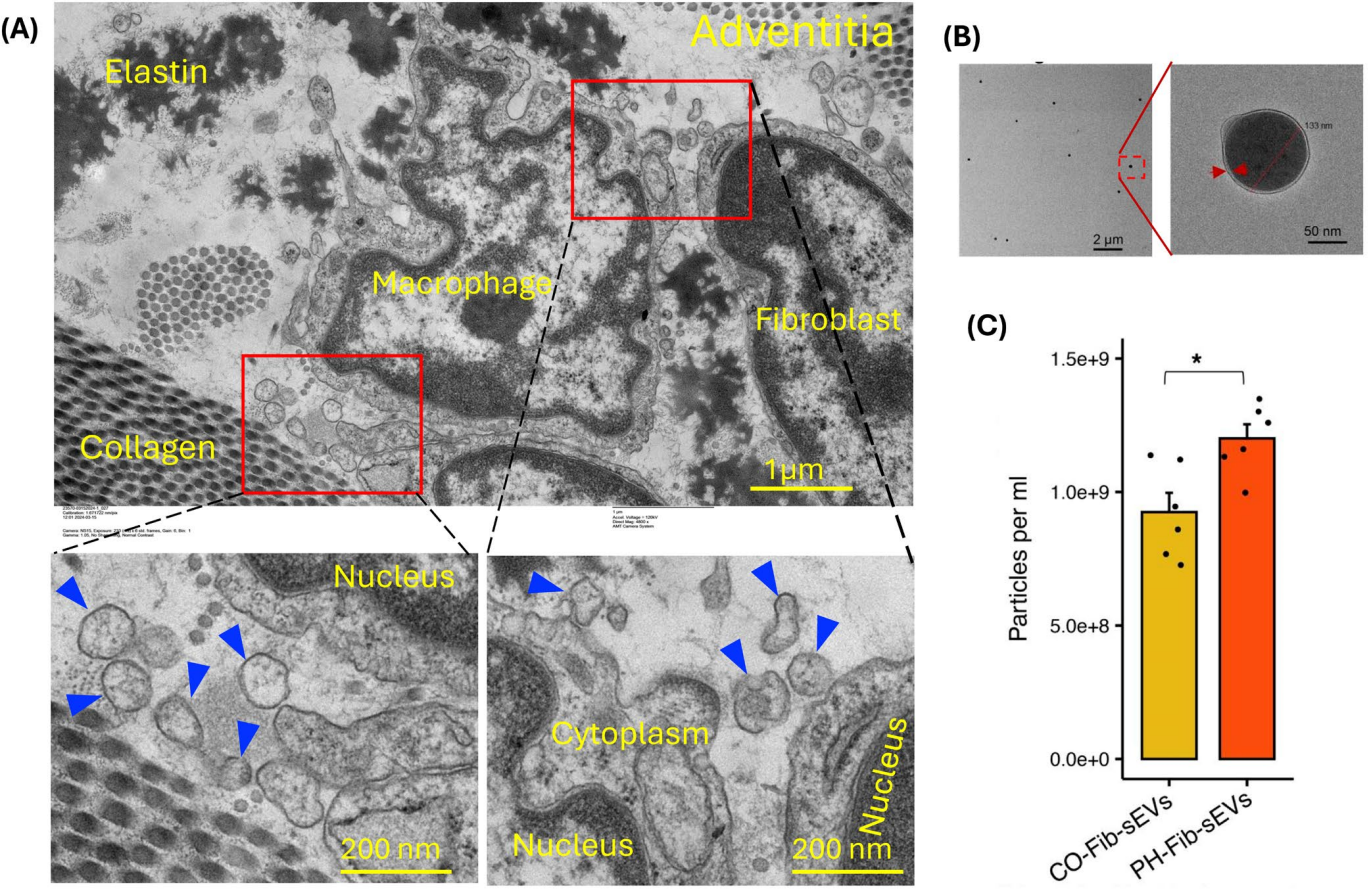
Extracellular vesicles in the adventitial microenvironment. (A) Transmission electron microscopy (TEM) of young‐calf precision‐cut lung slices (PCLS) showing the pulmonary artery adventitia. Fibroblast‐like and macrophage‐like cells are present within the adventitia and are surrounded by extracellular matrix components, including elastin and collagen fibers, as well as extracellular vesicles (EVs). Boxed regions (shown at higher magnification below) highlight EVs (blue arrowheads) within the adventitial microenvironment. Scale bars: 1 μm (top) and 200 nm (bottom). (B) Small extracellular vesicles (sEVs) isolated from adventitial fibroblast conditioned media. Transmission electron microscopy was used to evaluate the size and morphology of the vesicles. (C) Quantification of extracellular vesicles in conditioned media by nanoparticle tracking analysis (NTA) showed that PH‐Fibs secreted a greater number of sEVs than CO‐Fibs. (Adapted from ref. (Kumar et al. 2021)).

We recently demonstrated that small extracellular vesicles (sEVs) released by pulmonary vascular adventitial fibroblasts derived from PH animals are critical mediators of complement‐driven vascular inflammation and contribute to disease progression (Kumar et al. 2021). Compared with sEVs derived from control fibroblasts (CO‐Fib‐sEVs), PH‐Fib‐sEVs displayed a markedly distinct proteomic profile that promoted macrophage activation toward a proinflammatory and metabolically reprogrammed phenotype. Notably, we identified complement—and specifically the C3 component—as a key determinant of the capacity of sEVs to activate macrophages (Kumar et al. 2021). To assess these effects directly, we exposed naïve bone marrow–derived macrophages (BMDMs) to sEVs isolated from pulmonary vascular adventitial fibroblasts cultured from control and PH vessels. These studies revealed that (a) PH fibroblasts secreted significantly greater numbers of sEVs than control fibroblasts (Figure 10); (b) PH‐Fib–derived sEVs induced macrophage activation toward a proinflammatory and metabolically altered state, characterized by enhanced aerobic glycolysis and accumulation of succinate; (c) PH‐Fib‐sEVs exhibited a distinct proteomic signature relative to CO‐Fib‐sEVs, with pronounced enrichment of complement and coagulation proteins, consistent with the induction of proinflammatory and suppression of anti‐inflammatory pathways; and (d) sEVs derived from PH fibroblasts with depleted complement C3 elicited markedly attenuated proinflammatory gene expression and metabolic reprogramming in BMDMs. Recently we have verified sEVs within the adventitia of pulmonary artery using transmission electron microscopy of calf precision‐cut lung slices (Figure 10). Collectively, these findings indicate that complement‐containing sEVs secreted by pulmonary vascular fibroblasts from severely hypertensive animals play a critical role in shaping the local vascular microenvironment in PH.

Complement C3 is a central component of the alternative activation pathway and serves as a key convergence point for the classical and lectin pathways (Ricklin et al. 2016). Our observation that sEVs derived from C3‐depleted PH fibroblasts produced substantially diminished proinflammatory responses in BMDMs suggests that local synthesis of complement C3 by pulmonary perivascular fibroblasts and its subsequent packaging into sEVs provides a sufficient and functionally relevant source of complement to drive macrophage activation and metabolic reprogramming. These processes are likely to contribute to disease initiation and progression. Although the liver is the primary source of circulating complement proteins, including C3, our findings align with a growing body of literature demonstrating the importance of extrahepatic, locally produced complement in inflammatory and immune‐mediated diseases (Frid, McKeon, et al. 2020; Chan et al. 2019; Chmilewsky et al. 2014; Holers 2016; Lubbers et al. 2017).

## 4 Macrophages Amplify Inflammation and Perpetuate Vascular Remodeling

Macrophages are among the earliest and most prominent immune cells to infiltrate the pulmonary vasculature in PH and remain central to orchestrating chronic inflammation and remodeling. Both experimental and human studies consistently show marked perivascular macrophage accumulation, particularly within the adventitia and around plexiform lesions (Stenmark et al. 2013; Savai et al. 2012; Frid et al. 2006; Stenmark et al. 2015; Pugliese et al. 2017). These cells are recruited by chemokines (e.g., CCL2 and CCL5) and activated by complement components (e.g., C3a and C5a) produced by endothelial cells, pulmonary vascular smooth muscle cells, and especially activated fibroblasts. Macrophage function is tightly linked to metabolic reprograming (Li, Riddle, et al. 2021; El Kasmi and Stenmark 2015; Li et al. 2023; O'Neill and Pearce 2016). Hypoxia and inflammatory signaling activate HIF‐1α–dependent glycolysis, favoring rapid ATP generation and sustained cytokine production (Li, Riddle, et al. 2021; El Kasmi et al. 2014; Zuo et al. 2024). Mitochondrial dysfunction contributes to a pseudohypoxic, proinflammatory phenotype, while metabolites such as lactate and succinate further stabilize HIF‐1α and enhance IL‐1β expression. These interactions position macrophages as metabolic amplifiers of inflammation within the vascular niche. Upon activation by hypoxia, cytokines, and mechanical stress, macrophages undergo extensive phenotypic reprogramming. In experimental models where the trigger is evident, early PH disease is characterized by an acute inflammatory phase in which pro‐inflammatory macrophages dominate, with high production of IL‐1β, IL‐6, TNF‐α, and reactive oxygen species (ROS). These macrophages can amplify endothelial injury and recruit additional leukocytes (Pugliese et al. 2017; Zuo et al. 2024; Zhang, Wang, et al. 2023). As PH progresses, macrophages transition toward a pro‐remodeling phenotype that releases PDGF‐BB, TGF‐β, VEGF, and arginase‐1, driving fibroblast proliferation, collagen synthesis, and further cytokine release (Pugliese et al. 2017; Zuo et al. 2024; Zhang, Wang, et al. 2023). In complex disease states, these subsets coexist within the vascular wall, contributing both to tissue injury and maladaptive repair. Therapeutically, targeting macrophage recruitment or polarization can attenuate vascular remodeling. Inhibition of the CCL2/CCR2 axis reduces macrophage infiltration and right ventricular hypertrophy, whereas blockade of IL‐6 or TGF‐β signaling diminishes fibroblast activation and matrix expansion (Li, Riddle, et al. 2021; El Kasmi et al. 2014; Zuo et al. 2024). Collectively, macrophages integrate inflammatory, fibrotic, and metabolic pathways, acting as pivotal organizers of the innate immune response in PH.

## 5 T Cell Dysregulation Promotes Inflammation and Vascular Remodeling

**FIGURE 11:**
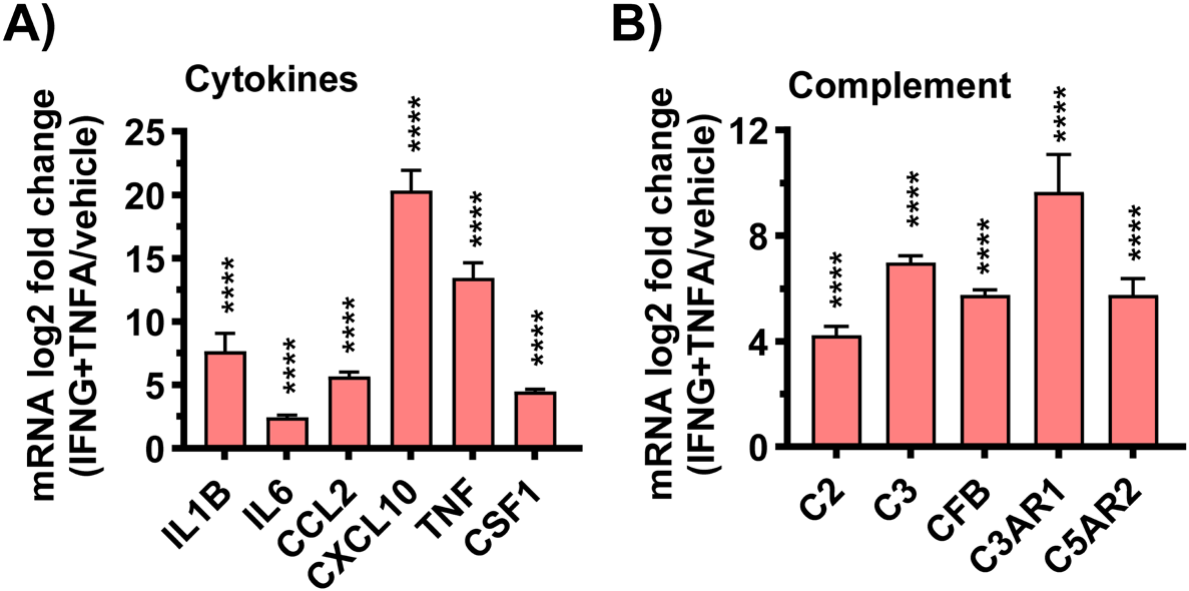
IFNG + TNFA drives cytokine and complement gene expression in IPAH fibroblasts. (A, B) IPAH fibroblasts were treated with vehicle or IFNG+TNFA (10 ng/mL of each) for 24 h and then cells were harvested for RNAseq analyses. The mRNA log_2_ fold change (IFNG+TNFA/vehicle) is shown for cytokines (A) and complement (B). Bar graphs represent mean ± SEM from *n* = 3 cell lines. Statistical significance was determined using the Wald test. ***p < 0.001, ****p < 0.0001.

T cells are prominently recruited to the remodeled PH vessels, particularly in the adventitia (Savai et al. 2012). T cells are broadly divided into CD4^+^ helper and CD8^+^ cytotoxic subsets, each with multiple specialized phenotypes that determine the nature and chronicity of inflammation. Experimental studies show that T cell deficiency completely abrogates hypoxia‐induced PH, whereas reconstitution with pro‐inflammatory CD4^+^ Th17 cells restores disease development (Maston et al. 2017). Paradoxically, certain T‐cell subsets exert protective effects. In the monocrotaline (MCT) and Sugen–hypoxia models, athymic (T‐cell–deficient) rats develop severe PH, which can be ameliorated by adoptive transfer of regulatory T cells (Tregs) (Tamosiuniene et al. 2011; Miyata et al. 2000; Taraseviciene‐Stewart et al. 2007; Ormiston et al. 2010). Tregs maintain immune tolerance and suppress excessive inflammation; their absence or dysfunction leads to unrestrained macrophage activation and fibroblast proliferation. These findings highlight the delicate balance between effector and regulatory T‐cell subsets in governing vascular inflammation. Human studies mirror these experimental observations. Lungs of IPAH patients show significant infiltration of both CD4^+^ and CD8^+^ T cells (Ferrian et al. 2024; Perros et al. 2012). Many of these cells exhibit an activated, memory phenotype and localize within the adventitia, where prominent interferon gamma (IFNG) production is observed and can induce proinflammatory macrophage and antigen‐presenting fibroblast phenotypes, sustaining a dysregulated adventitial microenvironment (Plecita‐Hlavata et al. 2023). In contrast, Treg deficiency removes a vital brake on inflammation, allowing fibroblast–immune interactions to dominate. Indeed, fibroblasts isolated from PH lungs, likely through their prominent expression of active complement fragements: C3a and C5a, promote differentiation of T cells toward a Th1 phenotype, while suppressing Treg markers such as FoxP3 (Plecita‐Hlavata et al. 2023). Reciprocally, GZMK CD8+ cells producing IFNg and TFNa can induce fibrobast‐production of cytokines, cytokine receptors and complement components that further drive the pro‐inflammatory and pro‐remodeling process, linking stromal–immune interactions to immune dsyregulation (Plecita‐Hlavata et al. 2023; Donado et al. 2025; Jonsson et al. 2022) (Figure 11).

## 6 Fibroblast–CD8 T Cell–Macrophage Cross‐Regulation in the Adventitial Niche

The data presented above support the concept that at the core of the adventitial inflammatory niche lies a pathogenic triad: fibroblasts, CD8 T cells, and macrophages. Fibroblasts act as early sentinels, sensing vascular injury and responding with proliferation, metabolic reprogramming, cytokine secretion, and complement production (Zhang et al. 2024; Chelladurai et al. 2022; Stenmark et al. 2015). Unlike control fibroblasts exposed to hypoxia, PH fibroblasts adopt a stable pathological phenotype marked by enduring epigenetic changes (Plecita‐Hlavata et al. 2023; Chelladurai et al. 2022; Plecita‐Hlavata et al. 2016). These cells release C3a, C5a, IL6, GM‐CSF, and CXCL12, driving recruitment and activation of macrophages and T cells (Figure 4). CD8 T cells, enriched in IPAH lungs (Perros et al. 2012) (Ferrian et al. 2024), reciprocate by secreting IFNG and GZMK. IFNG amplifies fibroblast complement production and induces macrophages to release IL1B and CXCL10. GZMK, as noted, directly activates complement and stimulates fibroblasts to produce inflammatory cytokines (Donado et al. 2025; Lan et al. 2025).

Macrophages, in turn, integrate signals from fibroblasts and T cells. They respond to C3a and C5a by producing IL1B and are primed by IFNG to secrete CXCL10, thereby perpetuating a cycle of inflammation and recruitment. Our studies, alongside others, have underscored macrophages as central mediators of experimental PH (El Kasmi and Stenmark 2015; Kumar et al. 2025; D'Alessandro et al. 2018; Stenmark et al. 2005). This three‐way dialogue results in a self‐reinforcing niche where complement, cytokines, and cellular interactions sustain inflammation. The persistence of this loop may explain why inflammation in PH does not resolve despite removal of the initial triggers, whether hypoxia, parasite infection, or hemolysis.

## 7 Adventitia and Adaptive Immune Response Beyond Macrophages and T Cells

A defining histological hallmark of adaptive immune involvement in PH is the formation of tertiary lymphoid structures (TLS), also termed tertiary lymphoid organs (TLOs), which closely resemble lymph nodes in their organization and function (Perros et al. 2012). These ectopic immune hubs, often located in the adventitial space around the remodeled pulmonary arteries, fibroblasts, contain B‐cell follicles, T‐cell zones, and antigen‐presenting dendritic cells, creating self‐sustaining centers of immune activation (Perros et al. 2012; Colvin et al. 2013). TLS in PH lungs not only facilitates local antibody production but also fosters B‐T‐cell interactions that drive class switching, affinity maturation, and the generation of long‐lived plasma cells. Importantly, blockade of TLS formation in experimental PH prevents disease development (Colvin et al. 2013), providing direct evidence that these structures are not mere bystanders but active participants in pathogenesis. Enrichment of TLS in the adventitial space around the remodeled pulmonary vasculature further sustains the theme that the adventitia serves as a command center for immune signal integration and regulation in PVD.

## 8 Adventitial Pro‐Inflammatory and Pro‐Remodeling Niches Across PVD

Although IPAH has served as the prototypical lens through which adventitial inflammation and complement biology are studied, it is only one piece of the broader PVD puzzle. By incorporating models across the disease spectrum, such as Schistosomiasis‐associated PAH (SchisPAH), sickle cell disease‐associated PH (SCD‐PH), systemic sclerosis, and heart disease, we can gain critical comparative insights into shared and divergent mechanisms across etiologies.

In SchisPAH, chronic parasite infection induces a strong Th2 immune response, with deposition of parasite eggs in pulmonary vessels driving granulomatous inflammation. Studies from Dr. Graham and colleagues have shown that CD4 T cells play a dominant role in establishing and sustaining the fibroblast‐rich adventitial niche. Complement contributes via lectin pathway activation, as mannose‐binding lectin engagement on T cells regulates disease progression (Kumar et al. 2024). The fibroblast‐T cell complement axis identified in IPAH thus finds a parallel in this infection‐driven model, albeit with distinct immunologic inputs.

In SCD‐PH, intravascular hemolysis and iron overload dominate the landscape. Free heme from sickled erythrocytes induces macrophage activation and iron deposition, which in turn accelerates adventitial remodeling and inflammation (Redinus et al. 2019). Here, the alternative complement pathway amplifies hemolysis‐induced injury, generating C3 and C5 fragments that fuel macrophage‐driven cytokine release. Studies have shown that iron‐overloaded macrophages exert profound effects on fibroblast phenotype, highlighting how metabolic and complement‐driven stress converge on the adventitial niche.

In autoimmune diseases, such as systemic lupus erythematosus (SLE), RA and SSc, there is dysregulation and inadequate control of the complement system with evidence of complement component activation and/or increased split products (C3a, C5a), which are thought to contribute to pathogenesis (Sturfelt and Truedsson 2012). Interestingly, it is also known that genetically regulated lack of certain complement proteins is associated with the development of autoimmune disease (Liphaus et al. 2015). In inflammatory arthritis, the complement cascade shows increased activity in response to immune complexes that form within the synovial tissue, leading to the recruitment of immune cells to the joint and contributing to the inflammatory milieu (Holers and Banda 2018). In addition, it has been recognized that intracellular complement, particularly in synovial fibroblasts, plays an important role in tissue priming (Friscic et al. 2021). Further, studies of acute inflammatory arthritis in complement deficient mice (C3, fB, and C5) have shown protection from collagen induced arthritis (Banda et al. 2007).

Pulmonary hypertension in SSc patients is characterized by pulmonary vascular remodeling and perivascular inflammation and is a leading cause of SSc morbidity and mortality (Chaisson and Hassoun 2013). SSc patients with pulmonary vascular disease have evidence of microvascular complement deposition and studies have shown that patients with SSc associated PH have elevated levels of complement factor‐D (Korman et al. 2017).

Recent studies using a tumor necrosis factor transgenic mouse model (TNF‐Tg: line3647) characterized by chronic arthritis and PH were used to study the effects of C3 and factor‐B knockouts to quantify effects on disease. Importantly, in this model and in distinct contrast to the other models, complement deficiency in a chronic inflammatory model does not ameliorate joint or pulmonary disease (Chen et al. 2026).

Although the role of complement in pulmonary hypertension associated with left heart disease (LHD‐PH) has not been directly examined, emerging evidence suggests that complement activation may play an important regulatory role in this condition. Complement activation, particularly dysregulation of the alternative pathway marked by elevated levels of CFB and CFD, has been implicated in the pathogenesis of LHD and correlates with disease severity. Moreover, the severe form of LHD‐PH, characterized by combined pre‐ and post‐capillary pulmonary hypertension (Cpc‐PH), shares genetic overlap with PAH and exhibits pulmonary vascular remodeling similar to that observed in PAH. Together, these observations raise the possibility that complement activation contributes to pulmonary vascular remodeling and disease progression in LHD‐PH and warrant future investigations (Shahini et al. 2017; Holt et al. 2021; Fayyaz et al. 2018; Assad et al. 2016).

Taken together, IPAH, SchisPAH, SCD‐PH, SSc, and left heart disease represent distinct entry points hypoxia, infection, and hemolysis into a common pathogenic architecture: fibroblast‐immune cell crosstalk governed by complement signaling. This convergence underscores the value of a comparative, integrative approach to uncover universal mechanisms and therapeutic targets.

## 9 Complement as a Unifying Driver

The collective data place complement at the heart of PH pathobiology. Across models, complement orchestrates the inflammatory niche by: (1) Activating fibroblasts into pro‐inflammatory, apoptosis‐resistant states. (2) Recruiting and polarizing immune cells through anaphylatoxins and EV‐mediated signaling. (3) Linking innate and adaptive immunity via novel pathways such as GZMK‐mediated complement activation. (4) Amplifying metabolic and stress responses, including iron overload and hypoxia‐induced inflammation.

The recognition that complement is enacted in multiple compartments‐plasma, extracellular microenvironment, intracellular complosome, and EV‐expands our mechanistic framework. Rather than a single linear cascade, complement in PH functions as a layered network of overlapping modules, each reinforcing the others to sustain inflammation and remodeling (Figure 7).

These mechanistic insights open new therapeutic avenues. Complement‐targeted agents are already in clinical development for other diseases, and recent studies highlight the therapeutic potential of complement inhibition in PAH.

### 9.1 Proximal Inhibition

C3 activation serves as the “central molecule” in complement‐mediated vascular remodeling, integrating inflammatory signals across the vessel wall's cellular landscape. Cleavage products C3a and C5a orchestrate massive immune cell recruitment, guiding neutrophils and monocytes to injury sites where they release pro‐inflammatory cytokines, creating a self‐sustaining loop of tissue damage (DeVaughn et al. 2024).

Furthermore, C3a induces a key phenotypic change in vascular smooth muscle cells (SMC), shifting them from a contractile to a synthetic, migratory state that contributes to intimal hyperplasia and thickening of the vessel wall (Ruan and Gao 2019; Han et al. 2012). In the adventitia, local or intracellular activation of C3 promotes fibroblast activation, speeding up ECM buildup and arterial stiffening (Kumar et al. 2024; Ruan et al. 2010). Strategies targeting C3, such as cyclic peptides like CP40‐KK, bind native C3 with high affinity, thereby blocking its proteolytic activation. CP40‐KK has shown improved solubility and a longer half‐life, successfully alleviating established PAH by reducing C3a‐driven NLRP3 inflammasome activation (Dai et al. 2024). Soluble CRIg‐Fc fusion proteins, which combine the extracellular domain of CRIg with an IgG Fc, bind C3b/iC3b, thereby inhibiting alternative pathway convertase formation and reducing inflammation in various preclinical models (Lieberman et al. 2015; Chen et al. 2011; Katschke Jr. et al. 2007). Factor H mimetics, such as mini‐FH and TT30, imitate the natural regulator Factor H to promote C3b breakdown, though their therapeutic use is limited by large size and potential immunogenicity (Risitano et al. 2012).

### 9.2 Alternative Pathway Inhibition

Since the expression of CFD and CFB is significantly increased in PH, targeting the alternative pathway provides a precise approach to reduce remodeling (Li et al. 2016; Kumar et al. 2024). Vemircopan (ALXN2050) is a potent, second‐generation oral inhibitor of CFD, developed for paroxysmal nocturnal hemoglobinuria (PNH). It is based on danicopan, the first‐in‐class FDA‐approved inhibitor used with ravulizumab or eculizumab to treat adults with PNH (Kulasekararaj et al. 2025). Vemircopan offers improved inhibition and pharmacokinetics, including higher bioavailability and lower clearance. In vitro studies show Vemircopan affecting MCP1, SDF1, IL‐33, and IL‐6 in isolated PH adventitial fibroblasts. Iptacopan, a strong oral CFB inhibitor, is an effective monotherapy for PNH, managing both intravascular and extravascular hemolysis. Beyond PNH, it is FDA‐approved for IgA Nephropathy and C3 Glomerulopathy because it reduces proteinuria and supports kidney function (Schubart et al. 2019). Since CFB expression is elevated in PH, a CFB inhibitor such as Iptacopan may offer a promising way to decrease inflammation and vascular remodeling in PH.

### 9.3 Terminal Inhibition

In PNH, intravascular hemolysis reduces nitric oxide (NO) levels, contributing to secondary PH. Eculizumab, a C5 complement inhibitor, significantly decreases cell‐free hemoglobin and NT‐proBNP levels. By preventing red blood cell breakdown, it restores NO availability and effectively relieves PH related to hemolytic conditions (Hill et al. 2010).

GZMK inhibition, though still in preclinical stages, offers a novel strategy to disrupt the fourth pathway linking CD8 T cells to complement activation (Donado et al. 2025; Turner 2025).

Beyond direct complement inhibition, therapies could aim to disrupt the fibroblast–immune dialogue itself. Targeting cytokine circuits (e.g., IL1B, CXCL10, IFNG) or blocking EV‐mediated complement transfer may attenuate the persistence of inflammatory niches. Importantly, these strategies must be tested not only in IPAH but also in other forms of PH such as SchisPAH and SCD‐PH, ensuring broad applicability across etiologies.

The translational potential of this approach is amplified by advances in precision spatial omics, which can serve as biomarkers of therapeutic response. Spatial transcriptomics including DSP allow direct readouts of how interventions reshape the adventitial landscape, providing mechanistic and diagnostic insights simultaneously.

## 10 Concluding Perspectives

PH has long resisted disease‐modifying therapy, in part because interventions targeted downstream hemodynamic consequences rather than upstream pathogenic processes. The recognition of adventitial fibroblast–immune–complement interactions as central to disease initiation and persistence reframes the field. What emerges is a picture of the adventitia as a dynamic, immunologically active environment, shaped by local complement activation across multiple compartments and amplified by a newly recognized GZMK‐dependent pathway (Figure 1).

Overall, this spatial convergence of immune cells, particularly macrophages and T cells, and fibroblasts, demonstrates that the adventitia serves as the central command center where fibroblasts, macrophages, and T cells interact to promote inflammation, vascular remodeling, and PH pathogenesis (Huertas et al. 2020; Stenmark et al. 2013; Tuder 2017; Zhang et al. 2024).

By uniting evidence from idiopathic, parasitic, and hemolysis‐driven forms of PH, a common paradigm becomes clear: diverse triggers converge on shared inflammatory circuits in the adventitia, sustained by complement activation and fibroblast–immune cell crosstalk. The applicability of the paradigm needs to be investigated in cardiopulmonary‐associated and other forms of PVD. However, based on the shared features of immune‐fibroblast convergence in the adventitial space and the complement's role in regulating immune balance, understanding how this paradigm across the spectrum of PVD will not only advance mechanistic understanding but also identify a rich landscape of therapeutic targets.

As the field moves forward, integrating cutting‐edge spatial transcriptomics, single‐cell profiling, and mechanistic animal models will provide the necessary resolution to unravel cellular heterogeneity and intercellular interactions. In parallel, the development of complement‐targeted therapies offers a timely opportunity to translate these insights into clinical benefit.

In sum, complement is not merely an accessory player in PH but a master regulator of the inflammatory niches that drive vascular remodeling and disease progression. By illuminating this role and charting new therapeutic strategies, the current program of work lays the foundation for a transformative shift in how PH is understood and treated.

## Author Contributions

All authors contributed equally to the preparation of this manuscript. Kurt R. Stenmark conceived the original idea and oversaw the overall project and manuscript development.

## Funding

This work was supported by the National Heart, Lung, and Blood Institute, P01HL152961, T32HL007171.

## Conflicts of Interest

The authors declare no conflicts of interest.

## Data Availability

The data that support the findings of this study are available from the corresponding author upon reasonable request.
